# Maternal Circulating Placental Growth Factor and Neonatal Metabolic Health Biomarkers in Small for Gestational Age Infants

**DOI:** 10.3389/fendo.2018.00198

**Published:** 2018-04-25

**Authors:** Hua He, Anne Monique Nuyt, Zhong-Cheng Luo, Francois Audibert, Lise Dubois, Shu-Qin Wei, Haim A. Abenhaim, Emmanuel Bujold, Isabelle Marc, Pierre Julien, William D. Fraser, 3D Study Group

**Affiliations:** ^1^Ministry of Education-Shanghai Key Laboratory of Children’s Environmental Health, Neonatology, Xinhua Hospital, Shanghai Jiao Tong University School of Medicine, Shanghai, China; ^2^Department of Obstetrics and Gynecology, University of Sherbrooke, Sherbrooke, QC, Canada; ^3^Lunenfeld-Tanenbaum Research Institute, Obstetrics and Gynecology, Mount Sinai Hospital, University of Toronto, Toronto, ON, Canada; ^4^Sainte-Justine Hospital Research Center, University of Montreal, Montreal, QC, Canada; ^5^School of Epidemiology, Public Health and Preventive Medicine, University of Ottawa, Ottawa, ON, Canada; ^6^Jewish General Hospital, McGill University Health Centre, Montreal, QC, Canada; ^7^Laval University Research Center, Quebec City, QC, Canada

**Keywords:** placental growth factor, small for gestational age, neonates, insulin, proinsulin, IGF-I, leptin, high-molecular-weight adiponectin

## Abstract

Small for gestational age (SGA) infants are at increased risk of type 2 diabetes in adulthood. It is unknown whether any prenatal biomarkers are helpful for identifying SGA infants with altered metabolic health profile at birth or later life. In a nested study of 162 SGA (birth weight < 10th percentile) and 161 optimal birth weight (25th–75th percentiles) control infants in the 3D (design, develop and discover) birth cohort in Canada, we assessed whether maternal circulating placental growth factor (PlGF), a biomarker of placental function, is associated with metabolic health biomarkers in SGA infants. Main outcomes were cord plasma insulin, proinsulin, insulin-like growth factor-I (IGF-I), leptin, and high-molecular weight (HMW) adiponectin concentrations. Maternal PlGF concentrations at 32–35 weeks of gestation were substantially lower in SGA versus control infants (*P* < 0.001), so as were cord plasma proinsulin (*P* = 0.005), IGF-I (*P* < 0.001), leptin (*P* < 0.001), and HMW adiponectin (*P* = 0.002) concentrations. In SGA infants with both low (<25th percentile) and normal maternal PlGF concentrations, cord plasma IGF-I and leptin concentrations were lower than control infants, but the decreases were to a greater extent in SGA infants with low maternal PlGF. Cord blood leptin levels were lower comparing SGA infants with low vs. normal maternal PlGF levels (*P* = 0.01). SGA infants with low maternal circulating PlGF levels at late gestation were characterized by greater decreases in cord blood IGF-I and leptin concentrations. Maternal circulating PlGF appears to be associated with neonatal metabolic health profile in SGA infants.

## Introduction

Placental growth factor (PlGF), a 50 kDa dimeric glycoprotein with a 132-amino acid residue, belongs to the vascular endothelial growth factor (VEGF) family and shares some biochemical and functional features with other VEGF family members (1, 2). It is expressed during early embryonic development, and prominent expression has been observed in the placenta throughout the gestation (2, 3). Trophoblast represents the major source of PlGF in the placenta (4). PlGF is an important local mediator of angiogenesis with a critical role in the modulation of vascular structure and function in the placental bed (3, 4). Downregulated expression of PlGF may cause placental vascular insufficiency, resulting in placental dysfunctional complications including preeclampsia, fetal growth restriction, or its surrogate indicator, small for gestational age (SGA) (5–10).

Fetal growth restriction or SGA has been consistently associated with increased risk of metabolic syndrome and type 2 diabetes mellitus in adulthood (11–13). However, only a proportion of SGA infants are destined to develop metabolic syndrome-related disorders. There is a lack of knowledge on which prenatal biomarkers may be useful for identifying SGA infants with altered metabolic health profile at birth or later life. To address this knowledge gap, we tested the hypothesis that maternal PlGF, a biomarker of placental function, may be associated with neonatal metabolic health profile in SGA infants, as indicated by cord blood concentrations of insulin, proinsulin, insulin-like growth factor-I (IGF-I), leptin, and high-molecular weight (HMW) adiponectin. These biomarkers are examined since their disturbances are often observed in metabolic syndrome-related disorders and type 2 diabetes in adulthood (14–17).

## Materials and Methods

### The 3D Birth Cohort, Data, and Specimens

This study was based on the recently described 3D (design, develop, and discover) birth cohort established by the Integrated Research Network of Perinatology in Quebec and Eastern Ontario (18). The 3D birth cohort is a large, carefully phenotyped birth cohort with linked biospecimen bank for studies on the associations between perinatal factors and infant health. Briefly, 2,366 women bearing a singleton fetus at 8–14 weeks of gestation were recruited from nine obstetrics hospitals in Quebec between May 2010 and August 2012. Exclusion criteria were as follows: (1) age < 18 or > 45 years; (2) illegal drug users; (3) severe illnesses or life threatening conditions; and (4) multiple pregnancies. The women were followed up at the second (20–24 weeks) and third (32–35 weeks) trimesters of pregnancy, and their infants were followed up at delivery. Data and specimens were collected at each study visit. All collected blood samples (including maternal and cord blood) were kept on ice, stored temporarily in a 4°C refrigerator and centrifuged within 30 min after the specimen collection. The separated plasma samples were stored in multiple aliquots in a freezer at −80°C until assays. The study was approved by the research ethics committees of Sainte-Justine hospital (the coordination center) (project no: 2899) and all participating hospitals. Written informed consent was obtained from all study participants.

### The 3D Cohort Nested SGA Study

**Figure 1:**
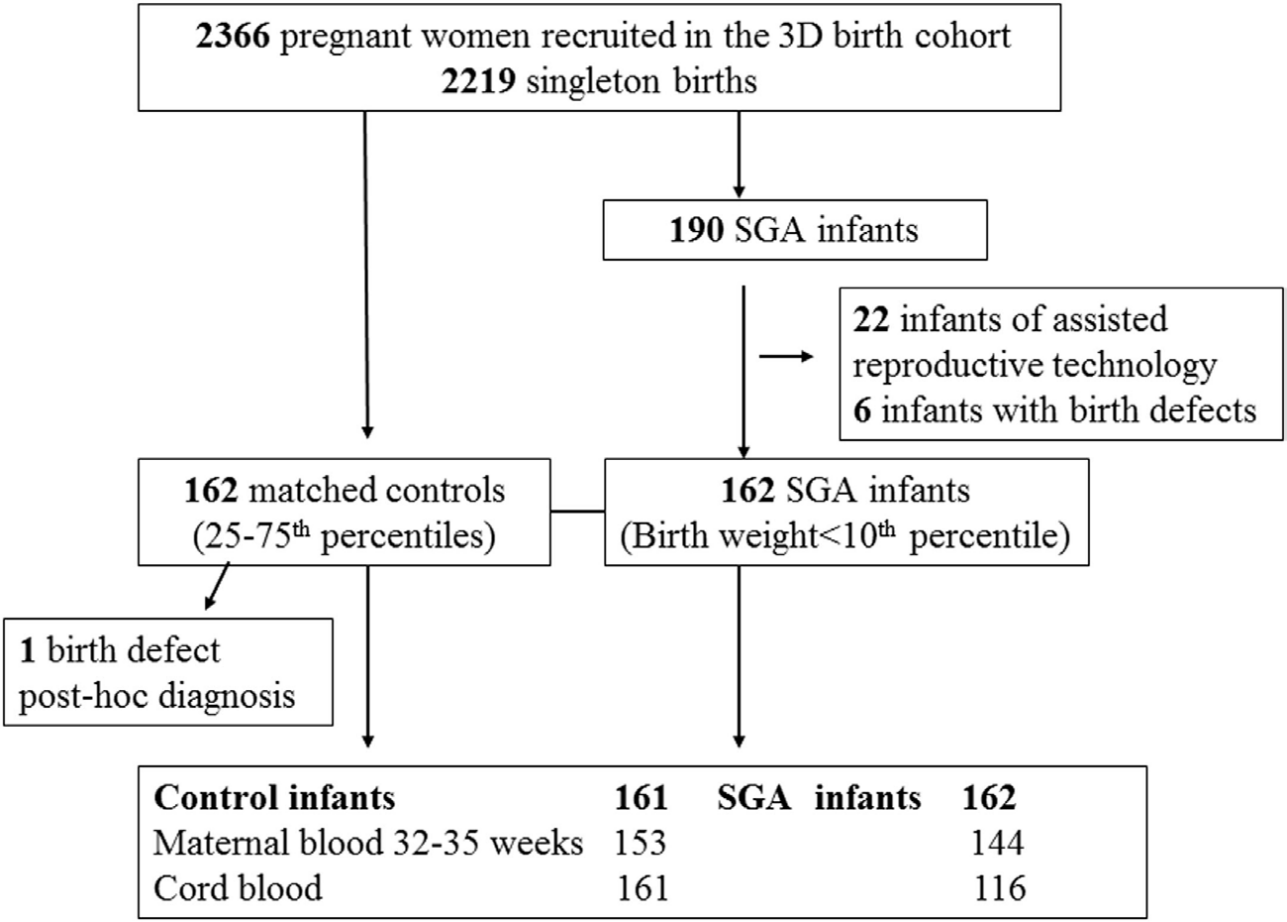
Selection of small for gestational age (SGA) and control study subjects in the 3D (design, develop, and discover) birth cohort.

We conducted a nested SGA study as part of the original 3D cohort research protocol. SGA was defined as birth weight < 10th percentile, according to the Canadian sex- and gestational age-specific birth weight standards (19). All study subjects (cases or control infants) must meet the following inclusion criteria: (1) singleton infants of mothers without severe pre-pregnancy illnesses (pre-existing diabetes, essential hypertension, etc.); (2) infants who were conceived without the use of artificial reproductive technology; (3) infants without any known birth defects; (4) gestational age at delivery >32 weeks. All SGA infants meeting the inclusion criteria were included (*n* = 162). Control subjects must further meet the following inclusion criteria: (1) optimal birth weight for sex and gestational age (between 25th and 75th percentiles); (2) cord blood specimen specimens available for biomarker assays. Each SGA infant was matched by a control infant according to maternal ethnicity (White, others), smoking status (current smoker, previous smoker, and no-smoker), and gestational age at delivery (33–36, ≥37 weeks). Each control was allowed to be a control for one SGA case only. All the controls were selected randomly among eligible subjects in the 3D cohort using a computing program written in Statistical Analysis System (SAS), Version 9.4. One control was excluded after biomarker assays since a birth defect was noted *post hoc*. Therefore, 162 SGA infants and 161 control infants constituted the final study sample. The selection of study subjects and availability of blood specimens for assays of maternal PlGF and cord blood biomarkers are illustrated in Figure 1.

### Outcomes

The main outcomes were neonatal metabolic health biomarkers including cord blood insulin, proinsulin, IGF-I, leptin, and HMW adiponectin concentrations. We measured HMW adiponectin since it is the major bioactive form of adiponectin with respect to its insulin sensitizing property and is the predominant form of adiponectin in cord blood (20).

### Biochemical Assays

Maternal plasma PlGF at 32–35 weeks of gestation was measured by a sensitive PlGF-specific antigen-capture enzyme-linked immunosorbent assay (R&D Systems, Minneapolis, MN, USA). We measured PlGF at 32–35 weeks of gestation since circulating PlGF levels peak during this period (6). Cord plasma glucose (in mmol/l, 1 mmol/l = 18 mg/dl) was determined by the automated glucose oxidase method (Beckman-Coulter, Brea, CA, USA), insulin (in pmol/l, 1 mU/ml = 6 pmol/l) by an automated ultrasensitive chemiluminescent immunometric assay (Beckman-Coulter, Brea, CA, USA), and proinsulin (pmol/L) by a quantitative ELISA kit (ALPCO Diagnostics, Salem, MA, USA) (21). Cord plasma total IGF-I was measured by an automated solid-phase, enzyme-labeled chemiluminescent assay after an acid buffer dilution to release the IGF-binding protein-bound IGF-I (22). Plasma leptin was measured by an ELISA kit (Invitrogen, Camarillo, CA, USA), and HMW adiponectin by a human HMW adiponectin ELISA kit (MyBioSource, San Diego, CA, USA). The inter-assay and intra-assay coefficients of variation of these assays were in the range of 2.0–9.6%.

### Statistical Analysis

Median, mean ± SD were presented for continuous variables. Log transformation was applied for biomarkers with skewed data distribution in correlation and regression analyses. Pearson partial correlation coefficients were calculated to assess the associations between maternal PlGF and metabolic health biomarkers in cord blood controlling for gestational age at blood sampling. Generalized linear models were applied to assess the associations controlling for covariables (potential confounders). The covariables in the adjustments included maternal age (<35 or ≥35 years), race (White, others), primiparity (yes/no), education (university: yes or no), smoking status (no, previous, current smoker), alcohol drinking (no, previous, current drinker), pre-pregnancy body mass index (BMI, kg/m^2^), gestational diabetes mellitus (yes/no), family history of diabetes (yes/no), gestational hypertensive disorder (yes/no), mode of delivery (cesarean/vaginal), infant sex (male/female), and gestational age at delivery (weeks). These variables were considered because they may affect cord blood metabolic health biomarkers. For the comparisons of cord blood insulin and proinsulin, the analyses were further adjusted for cord blood glucose (which affects insulin and proinsulin levels). Subjects with missing values were allowed to drop out in multivariate analyses. We did not adjust for birth weight in the regression models since birth weight may be considered an intermediate variable in the causal pathways to metabolic health. Also, birth weight is a variable to define SGA. The sample size is sufficient in the multivariate regression models since linear models require a minimal of only two subjects per variable for adequate estimation (23). Interactions between variables were assessed by testing the significance of interaction terms in generalized linear models. Data management and analyses were conducted using SAS version 9.4 (SAS Institute, Cary, NC, USA). Two-tailed *P* values < 0.01 were considered statistically significant, considering five primary outcomes of interest.

## Results

### Maternal and Infant Characteristics

Comparing SGA to control infants, there were no significant differences in maternal age, ethnicity, education, smoking, gestational diabetes, and family history of diabetes (Table 1). Women bearing a SGA infant had lower pre-pregnancy BMI (mean: 23.2 vs. 24.3 kg/m^2^) and were more likely to be primiparous (67.9 vs. 46.6%) and/or to have gestational hypertensive disorders (17.3 vs. 8.7%). SGA infants were more frequently delivered by cesarean section (26.5 vs. 17.4%). There were nine preterm deliveries in both SGA and control groups, all were mild preterm births (34–36 weeks of gestation).

**Table 1:** Maternal and infant characteristics of study subjects.

	SGA (*n* = 162)	Control (*n* = 161)	P*
**Mothers**			
Age (years)	30.7 ± 5.0	31.0 ± 4.5	0.54
≥35	36 (22.2)	28 (17.4)	0.28
Race, White	122 (75.3)	121 (75.2)	0.98
Primiparous	110 (67.9)	75 (46.6)	**<0.001**
Education, less than university	68 (42.8)	56 (35.4)	0.18
Smoking, current	41 (25.3)	41 (25.5)	1.00
Previous	28 (17.3)	28 (17.4)	
None	93 (57.4)	92 (57.1)	
Alcohol drinking, current	13 (8.4)	10 (6.5)	0.67
Previous	75 (48.4)	81 (52.9)	
None	67 (43.2)	62 (40.5)	
Marital status, not married/cohabited	13 (8.0)	6 (3.7)	0.26
Pre-pregnancy BMI (kg/m^2^)	23.2 ± 4.3	24.3 ± 5.1	0.036
Gestational diabetes	13 (8.1)	12 (7.5)	0.84
Gestational hypertensive disorder	28 (17.3)	14 (8.7)	**0.02**
Family history of diabetes	23 (14.2)	28 (17.4)	0.43
Prenatal glucocorticoids	6 (3.70)	7 (4.35)	0.78
**Infants**			
Cesarean delivery	43 (26.5)	28 (17.4)	0.047
Duration of labor (h)	10.5 ± 7.3	9.2 ± 7.7	0.072
Sex, male	85 (52.5)	70 (43.5)	0.11
Gestational age (weeks)	39.1 ± 1.5	39.0 ± 1.4	0.76
Preterm birth (<37 weeks)	9 (5.6)	9 (5.6)	0.99
Birth weight (g)	2,695 ± 298	3,370 ± 304	**<0.001**

*Data presented are *n* (%) or mean ± SD*.

*SGA, small for gestational age (<10th percentile in birth weight for sex and gestational age, according to the Canadian birth weight standards); BMI, body mass index*.

*P values in *t* tests for differences in means (for continuous variables) or Chi square tests for differences in proportions (categorical variables) between the two groups (SGA vs. control).

*P values in bold: P < 0.05*.

### Maternal PlGF and Cord Blood Metabolic Health Biomarkers

Adjusting for gestational age at blood sampling and maternal characteristics, circulating PlGF concentrations at 32–35 weeks of gestation were substantially lower in SGA compared to control infants (median, 444.1 vs. 825.6 pg/ml, adjusted *P* < 0.001) (Table 2). Adjusting maternal, pregnancy, and delivery characteristics, SGA infants had significantly lower cord blood concentrations of IGF-I (median, 4.1 vs. 8.0 nmol/l, *P* < 0.001), proinsulin (median, 9.0 vs. 12.4 pmol/l, *P* = 0.005), leptin (median, 7.5 vs. 9.7 ng/ml, *P* < 0.001), and HMW adiponectin (median, 17.1 vs. 20.2 µg/ml, *P* = 0.002) than control infants.

**Table 2:** Plasma concentrations of maternal blood PlGF (32–25 weeks of gestation) and cord blood biomarkers in SGA and control infants.

	SGA (*n* = 162)	Control (*n* = 161)	Adjusted *P*^a^
Maternal blood	(*N* = 144)^b^	(*N* = 153)^b^	
PlGF, pg/ml	444.1, 609.5 ± 481.2	825.6, 952.9 ± 643.8	**<0.001**
Cord blood	(*N* = 116)^b^	(*N* = 161)^b^	
Glucose, mmol/l	4.6, 4.9 ± 1.1	4.5, 4.5 ± 1.0	0.08
Insulin, pmol/l	23.4, 37.2 ± 43.2	28.8, 37.7 ± 29.9	0.11
Proinsulin, pmol/l	9.0, 13.5 ± 15.9	12.4, 16.4 ± 15.3	**0.005**
IGF-I, nmol/l	4.1, 4.2 ± 2.6	8.0, 8.1 ± 3.4	**<0.001**
Leptin, ng/ml	7.5, 8.9 ± 6.4	9.7, 11.8 ± 7.1	**<0.001**
HMW adiponectin, μg/ml	17.1, 18.0 ± 7.9	20.2, 20.8 ± 8.9	**0.002**

*Data presented are median, mean ± SD*.

*PlGF, placental growth factor; IGF-I, insulin-like growth factor-I; HMW, high-molecular weight; SGA, small for gestational age*.

*^a^*P* values for comparisons of mean values in log-transformed data between the two groups (SGA vs. control), adjusting for gestational age at blood sampling, maternal characteristics (age, parity, education, ethnicity, smoking, alcohol drinking, pre-pregnancy BMI, and family history of diabetes), and for all cord blood biomarkers further adjusting for pregnancy (gestational diabetes mellitus, gestational hypertensive disorder, and glucocorticoids use) and delivery (cesarean section, infant sex, gestational age, and duration of labor) characteristics, and for cord blood insulin and proinsulin, further adjusting for cord blood glucose. Data were log-transformed for biomarkers with skewed crude data distributions in the comparisons*.

*^b^The effective sample sizes in comparisons of biomarker data*.

*P values in bold: P < 0.01*.

### Correlations Between Maternal PlGF and Metabolic Health Biomarkers in Infants

There were no significant interactions between SGA status and maternal PlGF in relation to neonatal metabolic health biomarkers in infants (regression tests for interactions, all *P* > 0.1). Therefore, the correlations were reported for all study subjects together (rather than for SGA and control infants separately). Adjusting for gestational age at blood sampling and delivery (partial correlation analyses), maternal plasma PlGF at 32–35 weeks of gestation was positively correlated with birth weight (*r* = 0.35, *P* < 0.001) and cord blood IGF-I (*r* = 0.29, *P* < 0.001), leptin (*r* = 0.14, *P* = 0.03), and insulin (*r* = 0.13, *P* = 0.05). There were no correlations between maternal PlGF and cord blood glucose (*r* = 0.06, *P* = 0.36), proinsulin (*r* = 0.07, *P* = 0.29) or HMW adiponectin (*r* = −0.04, *P* = 0.48).

### Adjusted Associations

Adjusting for maternal and infant characteristics, each log unit increase in maternal plasma PlGF concentration was associated with a 19.6% (8.0–32.5%) increase in cord plasma IGF-I concentration (*P* < 0.001) and a 13.3% (1.3–26.8%) increase in cord plasma insulin concentration (*P* = 0.03), respectively (Table 3). Other associations were not statistically significant.

**Table 3:** Adjusted percentage change (95% CI) in birth weight and cord blood biomarkers in relation to per log unit increase in maternal plasma PlGF concentration at 32–35 weeks of gestation.

Adjusted% change^b^	Per log unit increase in maternal plasma PlGF	*P*^a^
Birth weight (*n* = 297)^c^	4.9 (3.1, 6.7)	**<0.001**
**Cord blood (*n* = 256)^c^**		
Insulin	13.3 (1.3, 26.8)	0.03
Proinsulin	3.8 (−6.8, 15.6)	0.50
IGF-I	19.6 (8.0, 32.5)	**<0.001**
Leptin	7.8 (−0.8, 17.2)	0.08
HMW adiponectin	−1.0 (−8.4, 6.9)	0.79

*^a^*P* values from generalized linear models adjusting for gestational age at blood sampling, maternal (age, parity, education, ethnicity, smoking, alcohol drinking, pre-pregnancy BMI, and family history of diabetes), pregnancy (gestational diabetes mellitus, gestational hypertensive disorder, and glucocorticoids use), and delivery (cesarean section, infant sex, gestational age, and duration of labor) characteristics, and for cord blood insulin and proinsulin, further adjusting for cord blood glucose concentration*.

*^b^The adjusted% change was calculated from the regression coefficient of the dependent variable (*y*) in log scale per log unit increase in the independent variable (*x*), because the regression coefficient (β) represents the proportion of change in *y* in the original scale: log *y*_1_ − log *y*_0_ = β, then log (*y*_1_/*y*_0_) = β, thus *y*_1_/*y*_0_ = *e*^β^, and thus the percentage change is (*e*^β^ − 1) × 100%*.

*^c^The effective sample sizes in regression models in estimating the adjusted% change in the dependent variable*.

*PlGF, placental growth factor; IGF-I, insulin-like growth factor-I; HMW, high-molecular weight; BMI, body mass index*.

*P values in bold: P < 0.01*.

### Metabolic Health Biomarkers in SGA Infants by Maternal PlGF

Table 4 presents cord blood metabolic health biomarkers in SGA infants stratified by maternal circulating PlGF concentration at 32–35 weeks of gestation: “low” (below the 25th percentile of control infants) or normal (≥25th percentile) in comparisons to control infants. SGA infants with either low (SGA1 subgroup) or normal (SGA2 subgroup) maternal circulating PlGF concentrations had lower cord blood IGF-I and leptin concentrations than control infants, to a greater extent in the SGA1 group (regression tests for trends across SGA1, SGA2, control groups, *P* < 0.001). Cord blood insulin concentrations were lower in SGA infants with low maternal PlGF (SGA1 group) relative to control infants.

**Table 4:** Birth weight and metabolic health biomarkers in cord blood comparing SGA infants with low (SGA1) or normal (SGA2) maternal circulating PlGF concentrations to control infants.

	SGA1 (*n* = 49)^b^	SGA2 (*n* = 54)^b^	Control (*n* = 153)^b^	Adjusted SGA1 vs. SGA2	*P*^a^	SGA2 vs. control
					SGA1 vs. control	
Birth weight, g	2,645, 2,607 ± 337	2,797, 2,770 ± 238	3,424, 3,370 ± 304	**0.001**	**<0.001**	**<0.001**
**Cord blood**						
Insulin, pmol/l	20.4, 29.6 ± 22.6	28.5, 43.7 ± 54.4	28.8, 37.7 ± 29.9	0.25	**0.008**	0.28
Proinsulin, pmol/l	8.7, 12.4 ± 11.5	9.3, 14.4 ± 19.0	12.4, 16.4 ± 15.3	0.79	0.046	**0.009**
IGF-I, nmol/l	3.9, 3.8 ± 2.2	4.2, 4.6 ± 2.8	8.0, 8.1 ± 3.4	0.18	**<0.001**	**<0.001**
Leptin, ng/ml	6.1, 7.3 ± 3.6	8.3, 10.2 ± 8.0	9.7, 11.8 ± 7.1	**0.01**	**0.0002**	0.02
HMW adiponectin, μg/ml	17.0, 17.4 ± 8.4	17.1, 18.6 ± 7.5	20.2, 20.8 ± 8.9	0.72	**0.01**	0.03

*Data presented are median, mean ± SD; biomarkers data were log-transformed in all comparisons*.

*SGA, small for gestational age (<10th percentile); SGA1, SGA with low maternal PlGF concentration (<25th percentile or 462 pg/ml); SGA2, SGA with normal maternal PlGF1 (≥25th percentile). PlGF, placental growth factor; IGF-I, insulin-like growth factor-I; HMW, high-molecular weight; BMI, body mass index*.

*^a^*P* values for comparisons of mean values in log-transformed data from generalized linear models adjusting for maternal (age, parity, education, ethnicity, smoking, alcohol drinking, pre-pregnancy BMI, and family history of diabetes), pregnancy (gestational diabetes mellitus, gestational hypertensive disorder, and glucocorticoids use), and delivery (cesarean section, infant sex, gestational age, and duration of labor) characteristics; for cord blood insulin and proinsulin, the comparisons were further adjusted for cord blood glucose concentration (which affects insulin and proinsulin concentrations)*.

*^b^The effective sample sizes for comparisons between groups in generalized linear models (subjects with data on both maternal PlGF and cord blood biomarkers)*.

*P values in bold: P ≤ 0.01*.

Comparing SGA1 vs. SGA2 infants, birth weights were significantly lower (*P* = 0.001), and cord blood leptin concentrations were lower (*P* = 0.01). There were no differences in cord blood IGF-1 and HMW adiponectin concentrations between SGA1 and SGA2 infants.

Comparing infants with low vs. normal maternal plasma PlGF in the control group, there were no significant differences in all observed cord blood biomarkers (Table 5).

**Table 5:** Birth weight and metabolic health biomarkers in cord blood comparing infants with low (<25th percentile) vs. normal maternal circulating PlGF concentrations within the control (birth weight 25th–75th percentile) group.

	Low PlGF (*n* = 34)	Normal PlGF (*n* = 118)	Adjusted *P*^a^
Birth weight, g	3,303, 3,228 ± 415	3,446, 3,414 ± 247	0.401
**Cord blood**			
Insulin, pmol/l	31.8, 39.0 ± 34.1	27.7, 37.3 ± 28.8	0.690
Proinsulin, pmol/l	12.9, 20.5 ± 26.8	12.0, 15.1 ± 8.9	0.294
IGF-I, nmol/l	7.7, 7.7 ± 3.5	8.0, 8.2 ± 3.4	0.752
Leptin, ng/ml	12.2, 13.7 ± 9.2	9.4, 11.2 ± 6.3	0.053
HMW adiponectin, μg/ml	19.9, 20.7 ± 9.3	20.7, 20.9 ± 8.8	0.761

*Data presented are median, mean ± SD*.

*^a^*P* values for comparisons of mean values in log-transformed data from generalized linear models adjusting for maternal (age, parity, education, ethnicity, smoking, alcohol drinking, pre-pregnancy BMI, and family history of diabetes), pregnancy (gestational diabetes mellitus, gestational hypertensive disorder, and glucocorticoids use), and delivery (cesarean section, infant sex, gestational age, and duration of labor) characteristics; for cord blood insulin and proinsulin, the comparisons were further adjusted for cord blood glucose concentration (which affects insulin and proinsulin concentrations)*.

*PlGF, placental growth factor; IGF-I, insulin-like growth factor-I; HMW, high-molecular weight; BMI, body mass index*.

There were no significant interactions between ethnicity or infant sex and SGA status affecting the observed associations.

The results were similar if the analyses were restricted to infants of mothers without gestational hypertensive disorders and gestational diabetes, or to White infants—the majority ethnic group (data not shown).

## Discussion

### Main Findings

To the best of our knowledge, the present study is the first to discover that SGA infants with low vs. normal maternal circulating PlGF levels at late gestation have greater decreases in cord blood IGF-I and leptin concentrations relative to optimal birth weight infants and that SGA infants with low maternal PlGF are characterized by lower leptin concentrations in comparison to SGA infants with normal maternal PlGF. These associations were robust and persistent even after adjusting for gestational hypertensive disorders, gestational diabetes, mode of delivery, gestational age, and other maternal and neonatal characteristics.

### Data Interpretation and Comparisons to Findings in Previous Studies

There is a lack of data on whether any perinatal biomarkers may be useful for identifying SGA infants with altered metabolic health indicators. PlGF is a placenta-derived angiogenic factor and a biomarker of placental function (3, 4). Circulating PlGF concentrations rise from the first to third trimesters of pregnancy, peak around 32 weeks of gestation, and decline in the last several weeks of pregnancy (6). Low circulating PlGF levels during early, mid, and late pregnancies are all strongly predictive of placental dysfunctional disorders including preeclampsia and SGA (5–10).

The present study extends our knowledge on the significance of maternal PlGF for neonatal metabolic health in infants. SGA infants with low maternal circulating PlGF concentrations in late gestation were observed to have greater alterations in fetal circulating concentrations of certain metabolic hormones (insulin, IGF-I, and leptin). SGA infants with low maternal PlGF levels had substantially lower cord blood leptin concentrations than SGA infants with normal maternal PlGF levels. The observations suggest that maternal PlGF may be a prenatal biomarker of neonatal metabolic health profile in SGA infants.

The mechanisms linking low maternal circulating PlGF levels with decreases in cord blood IGF-I and leptin levels are unclear. Low maternal PlGF levels are associated with placental insufficiency as indicated by Doppler measurement of uterine artery pulsatility index (24–26). Low maternal PlGF levels may be indicative of placental insufficiency with reduced nutrient transfer and oxygen supply across the placenta (4). Placental insufficiency may increase oxidative stress which affects a myriad of gene expression (27, 28). This may partly explain the alterations in cord blood levels of metabolic health biomarkers.

Consistent with the findings in previous studies, maternal circulating PlGF levels were positively associated with birth weight (8–10). Furthermore, the observed positive correlation between maternal circulating PlGF and cord blood IGF-I could provide a mechanistic explanation in the positive association between maternal PlGF and birth weight, because IGF-I is a major fetal growth factor strongly predictive of birth weight (22, 29).

The implications of neonatal hormones for postnatal metabolic health consequences are largely unknown. We are aware of only a few studies—all on the associations of cord blood adipokines with adiposity measurements in infancy, childhood, or adolescence. Cord blood adiponectin has been positively correlated with adiposity in infants at 1 month (30), in children at 3 years (31), and in adolescents at 17 years of age (32). We did not observe any difference in cord blood HMW adiponectin between SGA infants with low and normal maternal PlGF. The findings on cord blood leptin have been less consistent: it has been positively associated with adiposity at the age of 1 month (19), but negatively associated with adiposity in childhood (age 3 or 8 years) and early adolescence (age 13 years) in project Viva (31, 33, 34), yet positively associated with adiposity in childhood (age 9 years) and no association in adolescence (age 17 years) in a UK birth cohort study (32). Therefore, it remains unclear what the implications of low cord blood leptin in SGA infants with low maternal circulating PlGF for postnatal metabolic health consequences. There is a need for more studies on the implications of cord blood hormones for postnatal metabolic health consequences.

### Strengths and Limitations

The main strengths of the study are the prospective birth cohort with a relatively large number of SGA infants (*n* > 100), timely collection, and processing of blood specimens and high-quality biochemical assays. The main limitation is that we have only examined PlGF. One may speculate that if maternal PlGF is predictive of neonatal metabolic health biomarkers, other pro- and anti-angiogenic factors may also be useful and worth to be explored in future studies. The study was based on a Canadian birth cohort of largely White (Caucasian) subjects. More studies in other regions/populations are required to validate the generalizability of the findings to other populations.

## Conclusion

Small for gestational age infants with low vs. normal maternal circulating PlGF levels at late gestation were characterized by greater decreases in cord blood IGF-I and leptin concentrations. Maternal circulating PlGF appears to be associated with neonatal metabolic health profile in SGA infants.

## Ethics Statement

This study complies with the guidelines of the Declaration of Helsinki. The study was approved by the research ethics board of Sainte-Justine Hospital Research Center, University of Montreal. Written informed consent has been obtained from each study participant.

## Author Contributions

HH, Z-CL, AN, FA, LD, S-QW, HA, EB, IM, PJ, and WF contributed to study design and acquisition of research data. HH and Z-CL conducted the data analysis. HH drafted the manuscript. All authors contributed to improvements of the manuscript for important intellectual content and approved the final version for publication.

## Conflict of Interest Statement

The authors declare that the research was conducted in the absence of any commercial or financial relationships that could be construed as a potential conflict of interest.

## Abbreviations

HMW, high-molecular weight; IGF-I, insulin-like growth factor-I; PlGF, placental growth factor; SGA, small for gestational age.
